# Prolonged COVID-19 infection in a patient with B-cell acute lymphoblastic leukemia maintained on convalescent plasma until recovery with monoclonal antibodies

**DOI:** 10.1093/labmed/lmaf047

**Published:** 2025-07-26

**Authors:** Gagan Mathur, Jesse Qiao

**Affiliations:** University of California, Irvine, Orange, CA, United States; University of California, Irvine, Orange, CA, United States

**Keywords:** COVID-19, convalescent plasma, monoclonal antibodies, B-cell aplasia, immunocompromised host

## Abstract

**Introduction:**

Patients with hematologic malignancies who undergo immunosuppressive therapies such as chimeric antigen receptor T-cell (CAR-T) therapy are at high risk of prolonged SARS-CoV-2 infection due to impaired humoral immunity. Treatment options remain limited, with variable efficacy, in such settings.

**Methods:**

We describe a 21-year-old man with Down syndrome and B-cell acute lymphoblastic leukemia complicated by B-cell aplasia following CD19-directed CAR-T therapy. The patient developed COVID-19 and experienced persistent symptomatic infection, with high viral load and prolonged reverse transcriptase–polymerase chain reaction (RT-PCR) positivity for more than 7 months.

**Results:**

Despite multiple courses of remdesivir and extended weekly infusions of COVID-19 convalescent plasma (CCP), the patient remained viremic and intermittently symptomatic. Anti–SARS-CoV-2 immunoglobulin G titers were detectable only toward the latter time frame of treatment, and passive antibody therapy with CCP was insufficient for viral clearance. Ultimately, compassionate use of monoclonal antibody (mAb) therapy (casirivimab and imdevimab) was granted. Following administration, the patient achieved viral clearance for the first time, with resolution of symptoms and persistently negative RT-PCR findings for 8 months of available follow-up thereafter.

**Discussion:**

This case illustrates the limitations of CCP in patients with prolonged SARS-CoV-2 infection and highlights the effectiveness of mAbs in achieving viral clearance in severely immunocompromised hosts. It supports targeted use of mAb therapy in select high-risk populations and reinforces the importance of specific passive immunotherapy strategies (when available) for the management of viremia in immunodeficient patients.

## Introduction

The clinical spectrum of COVID-19, a respiratory illness caused by SARS-CoV-2, ranges from asymptomatic infection (approximately 40% of cases) to critical and fatal illness. Mild and uncomplicated COVID-19, which is seen in the majority (approximately 80%) of patients usually resolves within 2 weeks. Severe and complicated COVID-19 can have a natural course that runs for up to 3 months before complete recovery. In most patients with mild to moderate illness, viral shedding, as determined by culture, contact tracing, and subgenomic RNA detection, typically stops after 10 days from onset of symptoms. In rare cases with severe illness, prolonged viral shedding has been reported for up to 20 days from the onset of symptoms. Immunocompromised patients, however, who cannot mount a substantial antibody response to SARS-CoV-2, can have a lengthy disease course, with prolonged viral shedding and active viral replication lasting for months. Various treatment options available for management of COVID-19 include antiviral drugs (eg, remdesivir, molnupiravir, nirmatrelvir-ritonavir [Paxlovid; Pfizer]), immunomodulator drugs (eg, corticosteroids, interleukin 6 inhibitors, Janus kinase inhibitors), COVID-19 convalescent plasma (CCP), and anti–SARS-COV-2 monoclonal antibodies (mAbs). Here we present a case of a young patient undergoing therapy for B-cell acute lymphoblastic leukemia (B-ALL) who developed prolonged COVID-19 with active viral replication and shedding for more than 7 months after onset of symptoms. The patient was maintained on weekly CCP infusions and finally cleared SARS-CoV-2 infection with mAb treatment.

## Methods

### Patient characteristics

A 21-year-old male patient with Down syndrome (trisomy 21) with multiple comorbidities was diagnosed with B-ALL with central nervous system involvement. The patient was initially treated with combination chemotherapy but continued to have detectable residual disease at the end of the consolidation phase. Failed chemotherapy prompted initiation of CD19-directed chimeric antigen receptor T-cell (CAR-T) therapy tisagenlecleucel. The patient tolerated the CAR-T therapy well but continued to have pancytopenia due to chemotherapy. He was then discharged home with a baseline oxygen requirement of 0.5 L oxygen by nasal cannula for mild obstructive sleep apnea.

At a follow-up clinic visit 3 weeks after CAR-T therapy initiation, the patient had developed 2 days of cough after a possible sick contact exposure and subsequently tested positive on a nasopharyngeal swab specimen by reverse transcriptase–polymerase chain reaction (RT-PCR) for SARS-CoV-2 (day 0). On day 6, the patient presented to the emergency department with a fever and was admitted. His chest x-ray showed a diffuse interstitial prominence and a small bilateral pleural effusion. He continued to have intermittent fevers, with increased oxygen requirements over the next several days, including an episode of acute hypoxemic respiratory failure necessitating transfer to the pediatric intensive care unit with 10 L oxygen by face mask (day 18). During his pediatric intensive care unit stay, the patient received a 5-day course of remdesivir (200 mg intravenous [IV] on day 1, then 100 mg IV every 24 hours for 4 days). The patient showed clinical improvement; his oxygen requirement went down to 2 to 3 L by nasal cannula, and he was discharged home.

### COVID-19 disease course

A follow-up bone marrow biopsy showed no residual disease but did demonstrate B-cell aplasia (<0.1% CD19-positive lymphocytes). The patient was given IV immunoglobulin infusions for accompanying hypogammaglobulinemia. He continued to be symptomatic, with intermittent fevers, difficulty breathing, and oxygen requirements. He was admitted again on day 37 with increased oxygen requirements and bilateral patchy ground-glass opacities on chest computed tomography scans. He received a second 5-day course of remdesivir (200 mg IV on day 1, then 100 mg IV every 24 hours for 4 days) during this admission. By day 45, he showed some clinical improvement and was discharged home on his baseline oxygen requirement. The patient was readmitted on day 52 with persistent fevers and hypoxemia.

Throughout his disease course, the patient’s nasopharyngeal swab continued to test positive for SARS-CoV-2 by RT-PCR, and he had active viral shedding. Unfortunately, genomic sequencing of the viral isolate was not performed. No detectable anti–SARS-CoV-2 antibody titers (immunoglobulin G [IgG]) were identified during this time (Table I). It was assumed that due to B-cell aplasia, the patient could not mount a significant immune response to SARS-CoV-2 and thus could not clear it. By his fourth admission, it was decided to provide passive immunization to this patient with CCP. The goal was to prevent progression and potentially treat COVID-19 in this patient who had no antibody response of his own. The patient was given his first CCP infusion (2 units, approximately 200 mL each) on day 78. Anti–SARS-CoV-2 IgG titers were not assessed in CCP units before infusion because such assays had not yet been implemented as a standard practice across blood centers. The patient showed clinical improvement 3 days after the CCP infusion and was discharged home afebrile and on his baseline oxygen requirements.

**Table 1.** Delineation of COVID-19 testing Results (Semiquantitative PCR and IgG Results) With Corresponding Infusions Received

Date	PCR (Ct)	IgG (titer), before to after, with CCP given	Infusion
September 4, 2020	—	Negative (0.1)	CCP
September 11, 2020	—	Negative (0.2)	—
September 18, 2020	Positive (20.7)	Negative (0.1)	—
September 25, 2020	Positive (20.9)	—	—
September 29, 2020	—	Negative (0.2) to positive (1.6)	CCP (start weekly)
October 6, 2020	—	Negative (0.7) to positive (2.3)	CCP
October 13, 2020	Positive (19.3)	Borderline (0.9) to positive (1.0)	CCP
October 16, 2020	Positive (18.5)	—	
October 19, 2020	—	Borderline (0.9) to positive (5.8)	CCP
October 23, 2020	Positive (28.1)	—	
October 26, 2020	Positive (23.6)	Positive (3.3) to positive (3.7)	CCP
November 2, 2020	Positive (17.2)	Positive (2.5) to positive (2.2)	CCP
November 9, 2020	Positive (21.6)	Positive (2.5) to positive (2.4)	CCP
November 21, 2020	Positive (24.8)	—	—
November 30, 2020	—	Positive (1.6) to positive (1.6)	CCP
December 7, 2020	Positive (23.1)	Positive (1.7) to positive (1.8)	CCP
December 14, 2020	Positive (15.4)	Positive (1.2) to not done	CCP
December 21, 2020	Positive (20.4)	Positive (1.2) to positive (1.5)	CCP
December 29, 2020	Positive (20.6)	Borderline (1.0) to positive (1.1)	CCP
January 5, 2021	Positive (19.4)	Borderline (0.9) to borderline (1.0)	CCP
January 12, 2021	Positive (27.7)	Borderline (0.9) to borderline (0.8)	CCP
January 19, 2021	Positive (30.6)	Negative (0.7)	—
January 5, 2021	Positive (19.4)	Borderline (0.9) to borderline (1.0)	CCP
January 12, 2021	Positive (27.7)	Borderline (0.9) to borderline (0.8)	CCP
January 19, 2021	Positive (30.6)	Negative (0.7)	—
January 20, 2021	—	Positive (6.8)	Casirivimab plus imdevimab
January 25, 2021	Negative	Positive (6.9)	—
January 26, 2021	Negative	—	—
February 3, 2021	Negative	Positive (7.7)	—
February 10, 2021	—	Positive (6.9)	—
February 17, 2021	Negative	Positive (6.9)	—
March 1, 2021	Negative	—	—
March 10, 2021	Negative	—	—
May 25, 2021	Negative	—	—
July 24, 2021	Negative	—	—

Abbreviations: —, test not performed; CCP, COVID-19 convalescent plasma; CT, cycle time; IgG, immunoglobulin G; PCR, polymerase chain reaction.

## Results

The patient was readmitted 2 more times with fevers and continued pancytopenia after his first CCP infusion, and he continued to have positive RT-PCR results and negative IgG antibody titers. Weekly CCP therapy (2 units, approximately 200 mL each per week) was therefore initiated on day 103 to keep the viral infection under control and prevent hospital readmissions. Weekly CCP infusions were successful in keeping the patient relatively asymptomatic and out of the hospital for 4 weeks (days 103-144). Anti–SARS-CoV-2 IgG antibody testing was performed approximately weekly, both before and after each CCP infusion (Table 1). His anti–SARS-CoV-2 IgG titer became positive for the first time on day 103 after CCP infusion, but the patient continued to have a persistently positive SARS-CoV-2 RT-PCR result, with relatively high viral loads (cycle threshold = 17.2-28.1). A trial to change to biweekly CCP infusions (after infusion on day 144) failed, and the patient was readmitted on day 156 after he developed worsening cough, new fevers, and hypoxemia (oxygen saturation as measured by pulse oximetry = 80%) requiring 4 L oxygen by nasal cannula. Weekly CCP infusions were restarted on day 158 and appeared again to be successful in avoiding readmissions. Anti–SARS-CoV-2 IgG titers continued to stay relatively high with weekly CCP infusion, but the patient also continued to have positive RT-PCR results.

With no resolution in sight, these interventions continued until day 208, when mAb treatment was considered. Because the patient did not meet the emergency use authorization criteria for mAb therapy, a US Food and Drug Administration (FDA) Emergency Investigational New Drug application was obtained. The patient subsequently received mAb treatment (casirivimab and imdevimab [REGEN-COV]) on day 216.

**Figure 1.**
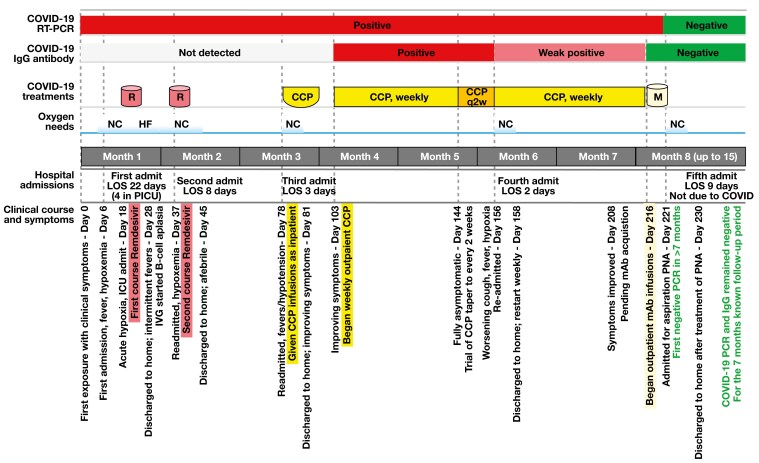
The timeline (drawn approximately to scale) shows the patient’s clinical presentation and hospital admission status in relation to COVID-19 test results, oxygen requirements, and treatments received for COVID-19. CCP indicates COVID-19 convalescent plasma; HF, high-flow oxygen mask, 10 L; IgG, immunoglobulin G; IVIG, intravenous immunoglobulin; LOS, length of stay; M, COVID-19 monoclonal antibody; mAb, monoclonal antibody; NC, nasal cannula; PICU, pediatric intensive care unit; PNA, pneumonia; q2w, every other week; R, remdesivir; RT-PCR, reverse transcriptase–polymerase chain reaction.

Incidentally, the patient was readmitted 5 days after mAb infusion with aspiration pneumonia, confirmed by chest x-ray on day 221. Notably, on that day, the patient finally tested negative for SARS-CoV-2 on RT-PCR since initial exposure 8 months prior. He eventually improved, with resolution of symptoms after 1 week of admission. He subsequently tested negative for SARS-CoV-2 on RT-PCR for a total of 10 separate and consecutive tests in the following 7 months. Figure 1 shows notable intervals of clinical course in relation to SARS-CoV-2 testing and treatments, depicted approximately to scale. Table 1 presents semiquantitative PCR results (cycle threshold values) and IgG antibody titers in relation to the treatments administered.

## Discussion

Immunocompromised patients, especially patients with hematologic malignancies, are at high risk of severe and prolonged SARS-CoV-2 infection, likely due to a suppressed ability to mount a substantial antibody response. An adverse side effect of CAR-T therapy for B-ALL is B-cell aplasia, as seen in our patient,^1^ resulting in the inability to clear infections, with prolonged disease course marked by prolonged viral shedding and active viral replication lasting for months. This effect increases the risk of spread as well as viral evolution and mutation. Persistent SARS-CoV-2 infection beyond 12 weeks is uncommon because most immunocompetent patients cease viral replication within 4 to 12 weeks.^2^ During long COVID syndromes, also known as postacute sequelae of SARS-CoV-2 infection, most patients do not have ongoing viral replication, and their COVID-19 tests (RT-PCR or antigen tests) are generally negative after the acute phase (typically within 2-4 weeks of symptom onset). This distinction highlights that the case reflects true ongoing viral replication rather than postacute sequelae. The patient’s 32-week duration of viremia is therefore exceptionally prolonged. It is therefore critical to continue to improve upon treatment strategies in an evolving pandemic. Therapies such as CCP and mAbs have been successfully employed in different situations. In a small case series of patients with B-cell malignancies treated with convalescent plasma,^3^ 5 of 10 untreated patients died, whereas 8 of 9 who received CCP became asymptomatic. In contrast, our patient remained intermittently symptomatic, despite extended CCP therapy, though not ill enough to require hospitalization or critical care. To our knowledge, this is the first reported case in which subsequent mAb therapy was successfully used to achieve viral eradication in a patient with B-ALL.

Although this patient is 1 of many throughout the pandemic to have received various therapies for SARS-CoV-2 infection, our case uniquely highlights persistent viremia, despite CCP and antiviral therapy. Between remdesivir, CCP, and mAbs, this patient showed definitive recovery only after receiving mAb treatment. This fact was evidenced by a negative RT-PCR test after 8 months of repeated positivity, along with resolution of respiratory symptoms and subsequent consistent RT-PCR negativity 7 months afterwards. Through his prolonged infection, CCP therapy was sufficiently beneficial to prevent hospital readmissions and occasionally detected IgG antibodies. Given the persistence of RT-PCR positivity and relatively high viral loads during his CCP therapy, however, the passively transfused IgG antibodies were likely not adequate in reducing the patient’s viral load. The persistently negative IgG titers before day 103 likely reflect impaired humoral immunity due to B-cell aplasia rather than a lack of CCP efficacy.

Notably, the timeline of this case coincided with the emergence and surge of the Alpha (and, to some extent, Beta) SARS-CoV-2 variants, before the widespread availability of vaccines. Although genomic sequencing was not clinically available at our institution, the patient’s prompt and sustained response to casirivimab plus imdevimab—mAbs known to be highly effective against the original and Alpha variants but substantially less effective against later strains—supports the inference that this was an Alpha-lineage infection rather than Beta, Gamma, Delta, or Omicron.^4,5^

### Convalescent plasma

Neutralizing antibodies are formed from infection and recovery or induced by vaccination. Convalescent plasma and mAbs passively provide neutralizing antibodies through direct infusion, and each has its own pros and cons. Passively transferred antibody levels peak immediately after infusion but decline markedly by day 4,^6^ whereas endogenous humoral and T-cell responses may persist up to and beyond 60 days.^7,8^ Convalescent plasma is limited by the collection and administration process because it requires willing donors and trained personnel for the infusion procedure. It also includes the known risks of transfusions: potential contamination with infectious agents, transfusion-associated reactions, and circulatory overload to the recipient.^9^ Yet these risks are considerably mitigated by following transfusion guidelines and proper protocols.^10^ A benefit of using CCP, the older therapy, is the diminished risk of unknown and long-term complications. Because CCP has been used in prior viral pandemics, such as the Spanish influenza outbreak in 1918, decades of collected data have contributed to its dependability.^11^

In COVID-19, CCP therapy offers potential benefits, particularly for immunocompromised patients and individuals with severe disease, by providing passive immunity and broad epitope recognition, which may enhance viral clearance and efficacy against emerging variants.^12,13^ Early administration appears more effective, but its overall benefit in the general population is limited.^14^ Variability in donor antibody titers, logistical challenges, and the risk of transfusion-related reactions further complicate the use of CCP.^13^ Given the polyclonal nature of CCP and the timing of donations during the predominance of the Alpha variant, the limited efficacy observed in our case was likely related more to dosing than to variant mismatch.

### Monoclonal antibodies

First licensed in 1986,^15^ mAbs offer a targeted therapeutic approach, but long-term effects remain uncertain. Cost considerations initially favored CCP, though mass production during the pandemic has rendered recombinant mAbs more cost competitive.^16^ The primary advantage of mAbs is their purified nature, which ensures target specificity and precise dosage control, a factor that likely contributed to our patient’s recovery.^17^ Studies worldwide support this hypothesis, showing viral clearance following mAb therapy in immunocompromised patients, including 2 UK cases treated with casirivimab plus imdevimab^18^ and a Czech multicenter study demonstrating reduced mortality in patients with hematologic malignancy receiving mAbs.^19^ Pertaining to the pediatric population, a multicenter retrospective study found mAb therapy to be effective.^20^ Comparative analysis of treated vs untreated cohorts further highlights improved clinical outcomes with mAb use.^21^

**Figure 2.**
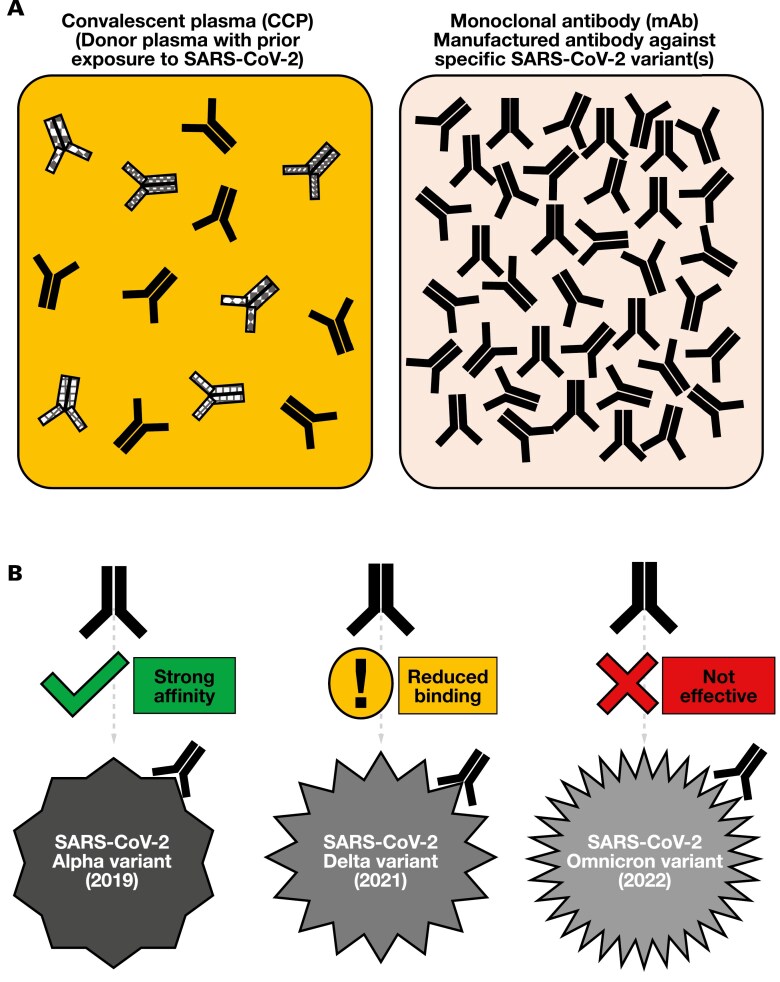
This illustration compares CCP and mAb therapy against COVID-19. The solid-color antibody, marked in the figure as
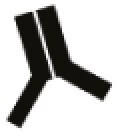
, denotes the antibody clone of highest specificity for the predominant SARS-CoV-2 variant at the time of plasma collection and manufacturing. (**A**) Differences in antibody content and concentration between CCP and mAbs. (**B**) Changes in viral structure (secondary to ongoing mutations, with the Alpha, Delta, and Omicron variants as examples) may affect antigen-antibody affinity, potentially affecting and reducing therapeutic efficacy. The shown antibodies are demonstrated for comparative purposes only and are not to actual proportion or exact molecular configurations. Sizes are also not drawn to scale. CCP indicates COVID-19 convalescent plasma; mAb, monoclonal antibody.

Similar to CCP and remdesivir, a notable disadvantage of mAbs is vulnerability to mutations that lead to escape variants, necessitating strategic use.^17^ Combination mAb therapies offer promise, though their broader application remains beyond this discussion. Epidemiologically, restricting passive therapies such as CCP and mAbs to high-risk, immunocompromised groups could help preserve their effectiveness. Although largely supplanted by mAbs, CCP may retain a role in future pandemics, particularly in the absence of newly developed targeted therapies. Figure 2 provides a visual representation of CCP vs mAbs, using an mAb against the Alpha variant as an illustration to highlight the concept of antibody specificity and effectiveness in viral neutralization.

### Current stance on COVID-19 mAbs and CCP

At the time of this case report, Regeneron’s mAb therapy, REGEN-COV, is no longer authorized for use against COVID-19 because of its reduced efficacy against the Omicron variant and subsequent strains.^22^ In January 2022, the FDA revoked the emergency use authorization for REGEN-COV, stating that it was “highly unlikely to be active against the Omicron variants.” In December 2024, the FDA formally revoked the authorization for REGEN-COV, along with other antibody treatments, citing their ineffectiveness against current variants and expired shelf life.^23^

Currently, high-titer CCP is recommended as a therapeutic option for COVID-19. A recent (January 2025) Association for the Advancement of Blood & Biotherapies bulletin announced the first blood center in the United States to provide FDA-licensed high-titer CCP.^24^ This development reflects ongoing efforts to provide standardized, high-potency CCP for immunocompromised patients who may benefit from passive antibody therapy, with FDA licensure reinforcing its role as a therapeutic option, especially when mAbs lose effectiveness against emerging variants.

Our report is limited by the absence of genomic sequencing and by the lack of standardized measurements for CCP neutralizing titers at blood donor centers. Despite these limitations, the temporal association between mAb administration and sustained RT-PCR negativity strongly supports its pivotal role in viral clearance.

Convalescent plasma provided sufficient clinical benefit to keep the patient from hospitalization, but the only treatment to show undetectable virus by RT-PCR was mAb therapy. Therefore, we propose the judicial use mAb therapy in the treatment of SARS-CoV-2, specifically for patients with immunodeficiencies secondary to hematologic malignancies, when available. If mAbs are not available or effective, high-titer CCP remains an alternative viable therapeutic option.
